# Recurrent Chromosomal Copy Number Alterations in Sporadic Chordomas

**DOI:** 10.1371/journal.pone.0018846

**Published:** 2011-05-13

**Authors:** Long Phi Le, G. Petur Nielsen, Andrew Eric Rosenberg, Dafydd Thomas, Julie M. Batten, Vikram Deshpande, Joseph Schwab, Zhenfeng Duan, Ramnik J. Xavier, Francis J. Hornicek, A. John Iafrate

**Affiliations:** 1 Department of Pathology, Massachusetts General Hospital, Harvard Medical School, Boston, Massachusetts, United States of America; 2 Department of Pathology, University of Michigan Health System, Ann Arbor, Michigan, United States of America; 3 Orthopaedic Oncology Service, Center for Sarcoma and Connective Tissue Oncology, Massachusetts General Hospital, Harvard Medical School, Boston, Massachusetts, United States of America; 4 Gastrointestinal Unit and Center for Computational and Integrative Biology, Massachusetts General Hospital, Harvard Medical School, Boston, Massachusetts, United States of America; 5 Broad Institute of MIT and Harvard, Boston, Massachusetts, United States of America; Health Canada, Canada

## Abstract

The molecular events in chordoma pathogenesis have not been fully delineated, particularly with respect to copy number changes. Understanding copy number alterations in chordoma may reveal critical disease mechanisms that could be exploited for tumor classification and therapy. We report the copy number analysis of 21 sporadic chordomas using array comparative genomic hybridization (CGH). Recurrent copy changes were further evaluated with immunohistochemistry, methylation specific PCR, and quantitative real-time PCR. Similar to previous findings, large copy number losses, involving chromosomes 1p, 3, 4, 9, 10, 13, 14, and 18, were more common than copy number gains. Loss of *CDKN2A* with or without loss of *CDKN2B* on 9p21.3 was observed in 16/20 (80%) unique cases of which six (30%) showed homozygous deletions ranging from 76 kilobases to 4.7 megabases. One copy loss of the 10q23.31 region which encodes *PTEN* was found in 16/20 (80%) cases. Loss of CDKN2A and PTEN expression in the majority of cases was not attributed to promoter methylation. Our sporadic chordoma cases did not show hotspot point mutations in some common cancer gene targets. Moreover, most of these sporadic tumors are not associated with *T* (brachyury) duplication or amplification. Deficiency of CDKN2A and PTEN expression, although shared across many other different types of tumors, likely represents a key aspect of chordoma pathogenesis. Sporadic chordomas may rely on mechanisms other than copy number gain if they indeed exploit T/brachyury for proliferation.

## Introduction

Chordoma is an uncommon malignant neoplasm with notochord differentiation that most often arises in the axial skeleton. The tumor is usually sporadic and rarely occurs as a familial case or a component of a syndrome. Chordoma has a long clinical course as it is typically slow growing; metastases tend to develop years after initial diagnosis. Regardless, it is often locally aggressive, and has a high rate of recurrence when not widely excised. Adequate excision is frequently difficult because of tumor proximity to the central nervous system and other vital structures.

Chordoma has a phenotype that recapitulates the notochord which is the precursor to and essential in the formation of the axial skeleton. Approximately 50% of chordomas arise in the sacrum, 35% in the skull base, and 15% in the mobile spine [1], while rare cases have been reported to originate in an extra-axial distribution or within soft tissues [2], [3]. Histologically, classic chordoma is composed of nests and cords of tumor cells with abundant eosinophilic or clear vacuolated cytoplasm and are enmeshed in abundant myxoid stroma. Recent studies based on biochemical analysis and immunohistochemistry have suggested that there may be a potential role for molecular therapy in the treatment of chordomas [4], [5], [6]. Although the morphology and immunoprofile of chordoma is well recognized, the genetic mechanisms underlying the development of the tumor have not been fully characterized. Understanding these processes is important as they govern the biological behavior of the neoplasm and may harbor potential relevant targets for therapy.

Karyotype analysis of chordomas has revealed several recurrent abnormalities. Losses of chromosome 1p and 3p short arms are frequently observed and implicated in the early development of the tumor [7], [8]. Chromosomal gains involving 7q, 20, 5q, and 12q, have been observed in at least 38% of cases studied although their relevance remains to be elucidated [8]. A few familial cases with linkage to 7q33 have been reported [9]. *T* (brachyury) gene duplication has been recently identified as a major susceptibility factor in familial chordoma by linkage analysis, high resolution array comparative genomic hybridization (CGH), and quantitative real-time polymerase chain reaction (PCR) [10]. Similarly, another study demonstrated *T* copy number gain in a fraction of sporadic chordomas and linked tumor proliferation to brachyury expression [11]. Interestingly, chordoma has been identified in patients with tuberous sclerosis complex (TSC) suggesting a possible role of the *TSC* gene in the pathogenesis of the disease [12]. Other specific defects implicated in chordoma include abnormalities in the retinoblastoma tumor suppressor gene [13], *p53* [14], and the gene for cyclin-dependent kinase inhibitor 2A and 2B [15]. The loss of the latter putative genes, *CDKN2A* and *CDKN2B*, was identified in 70% of classical chordoma biopsies evaluated with bacterial artificial chromosome (BAC) array CGH.

The application of non-biased, genome-wide approaches such as array CGH provides the greatest potential to discover unbalanced loci and candidate genes associated with disease which otherwise would not have been covered with low resolution and/or low throughput techniques such as karyotyping and FISH. To further understand the molecular pathogenesis of chordoma we utilized a genome-wide high-resolution oligonucleotide microarray to detect copy number changes in a set of 21 sporadic chordoma tissue specimens.

## Materials and Methods

### Patient data and tumor specimen

This study was conducted with the approval of the Massachusetts General Hospital Institutional Review Board (protocol 2008-P-000115/1; MGH) using anonymized, discarded clinical specimens. The study group consisted of 21 fresh frozen tumor tissue samples from histologically confirmed sporadic chordomas. Frozen section slides from each sample were generated and examined to substantiate the presence of adequate tumor cellularity prior to analysis.

### Array Comparative Genomic Hybridization Analysis

Genomic DNA was extracted from frozen tissue using the Gentra Puregene Blood Kit (QIAGEN, Valencia, CA). Genome-wide copy number alterations were analyzed by array comparative genomic hybridization using the Agilent 244K oligonucleotide array (Santa Clara, CA). The array contains more than 236,000 probes covering both coding and noncoding human sequences with an overall median probe spacing of 8.9 kb (7.4 kb in Refseq genes). Briefly, 0.5 micrograms (µg) of male human genomic control DNA (Promega, Madison, WI) and 0.5 µg of tumor DNA were digested with *Alu*I and *Rsa*I. Control and tumor DNA were labeled by random priming (BioPrime Array CGH Labeling Module, Invitrogen, Carlsbad, CA) with CY3- and CY5-dUTP dyes, respectively (GE Healthcare, Piscataway, NJ). The labeled DNA were purified with the Millipore Microcon YM-30 centrifugal filter device (Billerica, MA) and mixed in equal proportion for hybridization to the array in the presence of Cot-1 DNA (Invitrogen) using the Agilent Oligo CGH Hybridization Kit (Santa Clara, CA). Hybridization steps included 3 minutes denaturation at 95°C, pre-hybridization for 30 minutes at 37°C, and hybridization for 35 to 40 hours at 65°C. Following hybridization, the slides were washed with Agilent Oligo Array CGH Wash Buffer 1 and Buffer 2, at room temperature for 5 minutes and at 37°C for one minute, respectively. A final third wash was performed in stabilization and drying solution. Washed slides were scanned using the Agilent G2565 Microarray Scanner (Santa Clara, CA). Microarray TIFF (.tif) images were processed with Agilent's Feature Extraction Software v9.1 for data extraction. Array data was analyzed with the Agilent Genomic Workbench Standard Edition 5.0 software. Copy number aberration calls were made with a minimum regional absolute average log base 2 ratio of 0.25 and minimum contiguous probe count of 5. All array data were also manually reviewed for subtle copy number changes not detected by the software.

### CDKN2A (p16) Immunohistochemistry

Immunohistochemical studies were performed on formalin-fixed, paraffin-embedded tissue in all cases using the standard avidin-biotin-immunoperoxidase complex method. Prediluted p16 antibody was purchased from MTM Laboratories, Inc. (Heidelberg, Germany).

### PTEN Immunofluorescence

Double immunofluorescence staining was performed as previously described [16]. Briefly, after deparaffinization and rehydration, slides were subjected to microwave epitope retrieval in 7.5 mM sodium citrate buffer, pH 6. After rinsing several times in 10 mM Tris HCL buffer, pH 8 containing 0.154 M NaCl (TBS) supplemented with 0.05% (v/v) Tween-20 (TBST), endogenous peroxidase activity was blocked with 2.5% (v/v) H_2_O_2_ in methanol for 30 minutes. Non-specific binding of the antibodies was extinguished by a 30 minute incubation with “Background Sniper” (BioCare Medical, Concord, CA). The slide was then incubated with the tumor specific antibody, wide spectrum cytokeratin (DAKO, Carpinteria, CA, Z0622, Rabbit polyclonal antibody, 1∶250) overnight at 4C. The slides were washed with TBST twice for 5 minutes and then once with TBS for 5 minutes. The slides were incubated with the antibody to PTEN (Mouse monoclonal antibody, Novocastra, Newcastle, UK 1∶200) for 60 minutes at room temperature. Slides were then washed as described above and incubated with a combination of goat anti-rabbit IgG conjugated to AF555 (Molecular probes, Carlsbad, CA, A21424, 1∶200) in goat anti-mouse Envision+ (DAKO, Carpinteria, CA) for 60 minutes at room temperature in a dark humidity tray. The slides were washed as described above, and the target image was developed by a CSA reaction of Cy5 labeled tyramide (PerkinElmer, Waltham, MA, 1∶50). The slides were washed with 3 changes of TBS and stained with the DNA staining dye 4′, 6-diaminodo-2-phenylindole (DAPI) in a non-fading mounting media (ProLong Gold, Molecular probes, Carpinteria, CA). The slides were allowed to dry overnight in a dark dry chamber, and the edges were sealed.

The AQUA system (Software v2.2, HistoRx, New Haven, CT) was used for the automated image acquisition and analysis [16]. Briefly, images of each slide were captured with an Olympus BX51 microscope at 3 different extinction/emission wavelengths. Within each slide, the area of tumor was distinguished from stromal and necrotic areas by creating a tumor specific mask from the anti-cytokeratin stain, which was visualized from the Alexafluor 555 signal. The DAPI image was used to differentiate between the cytoplasmic and nuclear staining within the tumor mask. Finally, the fluorescence pixel intensity of the PTEN protein/antibody complex was obtained from the Cy5 signal with pixel intensity from 0–2000.

### SNaPshot Assay

A single base extension SNaPshot assay evaluating common point mutations in 13 cancer genes (*APC*, *BRAF*, *CTNNB1*, *EGFR*, *FLT3*, *JAK2*, *KIT*, *KRAS*, *NOTCH1*, *NRAS*, *PIK3CA*, *PTEN*, and *TP53*) was performed according to the manufacturer's protocol (Applied Biosystems (ABI), Foster City, CA). Tumor DNA was amplified with eight multiplex PCR reactions followed by treatment with exonuclease I and shrimp alkaline phosphatase (USBWeb, Cleveland, OH). The amplified DNA served as templates for multiplex single base primer extension. Genotyping primer extension products were analyzed with the ABI 3730 capillary electrophoresis system. Assay design and detailed protocol have been previously described [17].

### CDKN2A and PTEN Promoter Methylation Specific PCR

Purified genomic DNA from chordoma cases were bisulfite treated using the Zymo Research EZ DNA Methylation-Gold™. Promega Human Genomic DNA∶Male (Madison, WI) was used as the genomic unmethylated control. The same Promega genomic control DNA was globally methylated with *M. SssI* CpG Methyltransferase (New England Biolabs, Ipswich, MA) following the manufacturer's protocol to generate a methylated genomic DNA control. Following desulphonation and clean-up, DNA was eluted with 30 µL of the supplied elution buffer. Bisulfite treated DNA (2 µL) was analyzed with methylated or unmethylated specific primers by PCR (5 pmol of forward [Forw] and reverse [Rev] primers, 200 µM dNTP, 1.2 mM MgCl_2_, and 0.5 U Platinum *Taq* Polymerase in 10 µL total reaction volume; all reagents from Invitrogen, Carlsbad, CA). *CDKN2A* promoter analysis was performed with the following two previously reported primer sets: *p16*-M Forw TTATTAGAGGGTGGGGCGGATCGC, *p16*-M Rev GACCCCGAACCGCGACCGTAA, *p16*-U Forw TTATTAGAGGGTGGGGTGGATTGT, *p16*-U Rev CAACCCCAAACCACAACCATAA, *p16*-M2 Forw TTATTAGAGGGTGGGGCGGATCGC, *p16*-M2 Rev CCACCTAAATCGACCTCCGACCG, *p16*-U2 Forw TTATTAGAGGGTGGGGTGGATTGT, and *p16*-U2 Rev CCACCTAAATCAACCTCCAACCA [18]. In our study, the first *p16*-M and *p16*-U set is designated as *CDKN2A* MSP1 while the second *p16*-M2 and *p16*-U2 set is designated as *CDKN2A* MSP2. *PTEN* promoter analysis was performed with the following two previously reported primer sets: *PTEN*-I-M Forw TTTTTTTTCGGTTTTTCGAGGC, *PTEN*-I-M Rev CAATCGCGTCCCAACGCCG, *PTEN*-I-U Forw TTTTGAGGTGTTTGGGTTTTTGGT, *PTEN*-I-U Rev ACACAATCACATCCCAACACCA, *PTEN*-III-M Forw GGTTTCGGAGGTCGTCGGC, *PTEN*-III-M Rev CAACCGAATAATAACTACTACGACG, *PTEN*-III-U Forw TGGGTTTTGGAGGTTGTTGGT, and *PTEN*-III-U Rev ACTTAACTCTAAACCACAACCA [19]. In our study, the first *PTEN*-I-M and *PTEN*-I-U set is designated as *PTEN* MSP1 while the second *PTEN*-III-M and *PTEN*-III-U set is designated as *PTEN* MSP2. PCR touchdown thermocycling conditions were as follows: 95°C 5 minutes, [94°C 30 sec, 66°C 30 sec, 72°C 45 sec]×2 cycles, [94°C 30 sec, 64°C 30 sec, 72°C 45 sec]×2 cycles, [94°C 30 sec, 62°C 30 sec, 72°C 45 sec]×2 cycles, [94°C 30 sec, 60°C 30 sec, 72°C 45 sec]×34 cycles, 72°C 10 minutes. PCR products were separated by 1.25% agarose gel electrophoresis and visualized with ethidium bromide staining.

### T (Brachyury) Quantitative Real-Time PCR

Purified genomic DNA from chordoma cases were analyzed by Taqman quantitative real-time PCR in triplicate using two sets of PCR primers/probes, one targeting the *T* (*Brachyury*) gene on chromosome 6 and the other targeting the *MCM7* gene on chromosome 7 as a reference control. The primer sets are as follows: *T* Forw 5′-TCAGGAGTCAGAGTGCAGGA-3′, *T* Rev 5′-CGGACCAGGATGAGAGAGAG -3′, *T* Probe 5′-[6-FAM]CGGCAGCATTTGTTGGGAGAAACG[Tamra]-3′, *MCM7* Forw 5′-CGTGAGTGGAGAACTGACC-3′, *MCM7* Rev 5′-CAGCCATCTTGTCGAACTC-3′, and *MCM7* Probe 5′-[6-FAM]TGACCAGGGTGTGTGCTGCA[Tamra]-3′. Reaction conditions include 5 µL of DNA (diluted to approximately 5 ng/µL) or genomic standards, 1.67 pmol each of forward/reverse primers and probe, 200 µM dNTP, 1.2 mM MgCl_2_, and 0.5 U Platinum *Taq* polymerase in 10 µL total reaction volume (all reagents from Invitrogen). Promega Human Genomic DNA∶Male was serially diluted 1∶2 to make the genomic standards (20, 10, 5, 2.5, 1.25, 0.63, 0.31, and 0.16 ng/µL or equivalently 6080, 3040, 1520, 760, 380, 190, 95, and 48 copies/µL). PCR was performed with the Applied Biosystems 7500 Real-Time PCR System using the following thermocycling conditions: 95°C 2 minutes, [94°C 15 sec, 60°C 60 sec]×45 cycles. Ten non-chordoma blood DNA samples were used to establish a normal reference *T*∶*MCM7* ratio. The test samples were normalized to the normal reference ratio and further corrected for *MCM7* copy number status based on the array CGH data and estimated tumor percentage based on histological review. Finally, the normalized and corrected *T*∶*MCM7* ratios were multipled by a factor of 2 to obtain the absolute estimated *T* copy number.

### Statistical Analysis

Statistical analyses were performed with the XLSTAT software (version 2010.3.01; New York, NY). Genetic alterations were compared between different groups and conditions by the two-tailed unpaired Student's *t*-test and Fisher's exact test where appropriate. Chordoma cases were evaluated by k-means clustering based on quantitative PTEN immunofluorescence, assuming two classes (positive and negative staining groups) and using random initial partition. The Mann-Whitney *U* test was used to confirm the segregation result from k-means clustering. *P*-values of 0.05 or less were considered significant.

## Results

### Chordoma Cases

Frozen tumor specimens were obtained from 20 patients including 14 males and 6 females who ranged in age from 41 to 83 (median 61.5) years. All tumors were classified as conventional, sporadic chordomas by light microscopy except for one neoplasm which was diagnosed as a chondroid subtype (CH9). Seventeen samples were from the primary tumor and were located in the sacrum (10 cases), clivus (2 cases), and the mobile spine (5 cases). Four tumors were from local recurrences in the lumbar (CH6 and CH39) and sacral spine (CH34 and CH37). Both CH34 and CH37 were recurrent tumors from the same patient; the latter recurrent CH37 tumor was excluded from all statistical analyses. The majority of patients were treated with local radiation therapy (15 out of 20 cases) and followed for disease survival since the time of diagnosis. Nine patients died with an average survival of 8.2 years (range of 6–10 years, Table 1). No correlation was noted between survival and various characteristics of the tumor or patient, including age, gender, tumor location, tumor histology, and radiation treatment.

**Table 1:** Clinical information.

Case	Age	Gender	Location	Histology	XRT	Followup	Survival
CH1	70	M	Sacrum	Conventional	Y	A / NED	N/A
CH2	53	M	Clivus	Conventional	Y	A	N/A
CH3	75	F	Lumbar	Conventional	Y	D	11
CH5	73	M	T11	Conventional	N	A	N/A
CH6	41	M	Lumbar (recurrent)	Conventional	N	D	6
CH7	58	F	Clivus	Conventional	Y	D	10
CH8	60	F	Sacrum	Conventional	Y	A / NED	N/A
CH9	45	F	Cervical	Chondroid	Y	A	N/A
CH14	71	M	Sacrum	Conventional	Y	A / NED	N/A
CH30	80	M	Sacrum	Conventional	Y	A / NED	N/A
CH33	57	M	Lumbar	Conventional	Y	A	N/A
CH34	73	M	Sacrum (recurrent)	Conventional	Y	D / NED	10
CH35	71	F	Cervical	Conventional	Y	D / NED	8
CH36	52	F	Sacrum	Conventional	Y	A	N/A
CH37	74	M	Sacrum (recurrent)	Conventional	Y	D / NED	10
CH39	52	M	Lumbar (recurrent)	Conventional	Y	D	11
P527	77	M	Sacrum	Conventional	N	D	6
P554	55	M	Sacrum	Conventional	N	A	N/A
P937	46	M	Sacrum	Conventional	N	A	N/A
P984	83	M	Sacrum	Conventional	Y	D	6
P1033	63	M	Sacrum	Conventional	Y	D	6

XRT = Radiation therapy. A = Alive. D = Dead. NED = No evidence of disease. Survival in years from time of diagnosis. N/A = Not applicable. Note that cases CH34 and CH37 are recurrent tumors from the same patient.

### Array CGH

**Figure 1:**
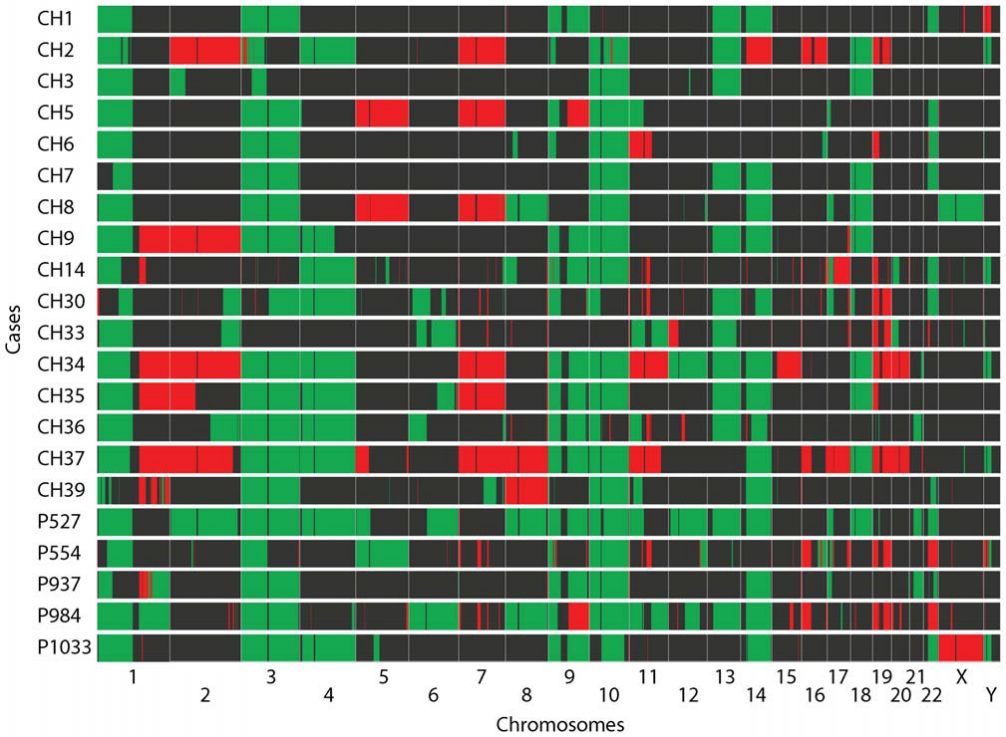
Heat map of array CGH results. Copy number gains (red) and losses (green) are displayed for each individual chordoma case (rows) with chromosomes organized in columns (separated by white vertical lines) and indicated by labels at the bottom. Note that cases CH34 and CH37 are recurrent tumors from the same patient.

**Figure 2:**
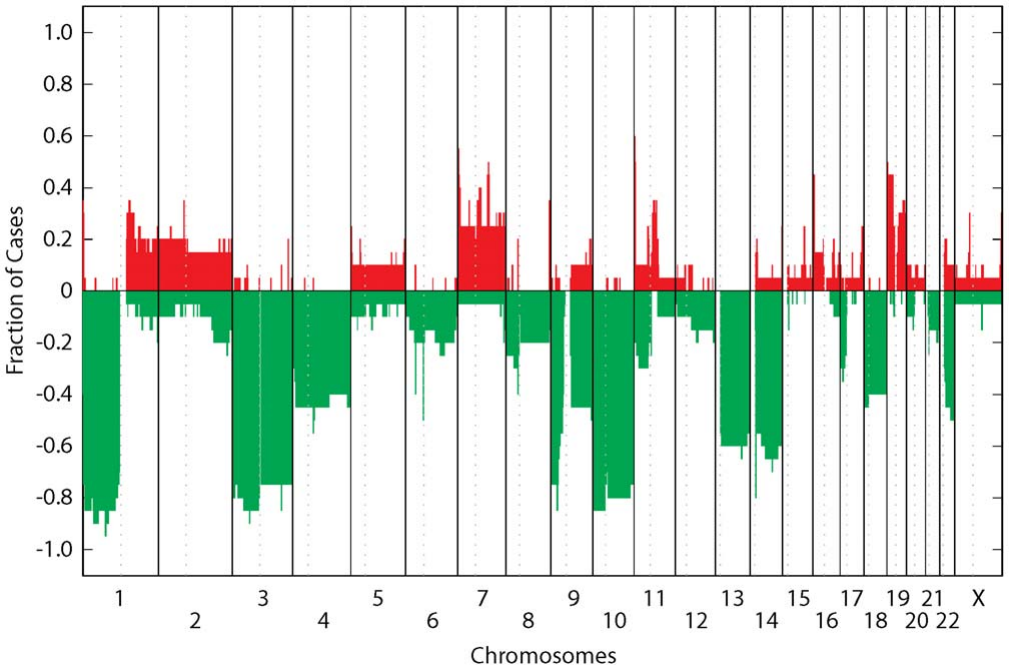
Frequency plot by genomic position. Array CGH data from all chordoma cases from 20 unique patients were combined and presented as copy number gain/loss frequencies relative to chromosome and genomic position. Note that chromosome Y is not depicted. The second recurrent CH37 tumor was excluded from analysis.

Comparative genomic hybridization using the Agilent 244K genome-wide oligonucleotide array showed predominantly copy number losses involving an average of 26.5±10.0% of the genome per case (16.5% to 56.6% range, excluding the Y chromosome, Figures 1 and 2). Copy number gains affected on average 7.1±7.2% of the genome per case (<0.1% to 27.1% range, excluding the Y chromosome), which is significantly less than involvement by copy number losses (*P*<0.01, Figures 1 and 2). Frequent whole chromosome changes included losses of chromosomes 3 (75%), 4 (40%), 9 (45%), 10 (75%), 13 (55%), 14 (55%), and 18 (40%). The most common chromosome gain involved chromosome 7 which was found in a total of 5 cases (25%).

Subchromosomal changes are displayed in Figure 1. Detailed maximal ranges and subranges of the involved regions are listed in Table 2. Of note, entire and partial loss of 1p was found in all cases, including three cases with partial telomeric 1p loss (CH14, CH39, P937) and three cases with partial centromeric 1p loss (CH7, CH30, P554). Loss of 10q, either through entire chromosome 10 loss or 10q loss only was evident in 16 of 20 cases (80%).

**Table 2:** Recurrent genomic changes in sporadic chordoma.

Cytogenetic Locus	Gain/Loss	Maximum Range	Subrange	Frequency	Candidate Genes
1p36.32-p11.1	Loss	0.74–121.05	0.55–52.39	0.85	*RUNX3*
			52.39–72.81	0.80	
			72.81–121.05	0.80	
3p29-p26.3	Loss	0.04–199.32	0.04–90.39	0.75	*MLH1*, *VHL*
			95.07–196.78	0.75	
			0.04–196.78	0.70	
4p16.3-q35.2	Loss	0.04–191.13	9.58–191.13	0.40	
6q21-q22.33	Loss	113.47–127.54	113.47–127.54	0.25	
7p36.3-p22.3	Gain	0.14–158.81	0.14–158.81	0.25	
			99.53–100.68	0.50	
			0.15–2.99	0.55	
			70.77–76.05	0.45	
8q24.3	Gain	142.70–145.79	142.70–145.79	0.35	
9p24.3-q34.3	Loss	0.21–140.15	0.21–140.15	0.25	*TSC1*, *PTCH1*
			0.23–38.81	0.55	*CDKN2A*, *CDKN2B*
10p15.3-q26.3	Loss	0.12–135.25	0.12–135.25	0.65	*PTEN*
			2.04–120.08*	0.80	*PTEN*
11p15.5-p11.12	Loss	0.18–50.64	0.18–50.64*	0.30	*WT1*
11q12.2-q13.4	Gain	60.24–72.78	60.24–72.78*	0.30	*MEN1*
13q11-q34	Loss	18.07–114.12	18.07–114.12*	0.60	*RB*, *BRCA2*
14q11.1-q32.33	Loss	18.62–106.35	18.62–106.35*	0.65	
17p13.3-p11.1	Loss	0.06–22.14	0.06–22.14*	0.35	*P53*, *NF1*
17q11.1-q25.3	Gain	22.81–78.65	71.85–78.05	0.25	*HER2*
18p11.32-q23	Loss	0.06–76.11	0.06–76.11	0.40	*SMAD4*
19p13.3-q13.43	Gain	0.21–63.78	0.21–63.78*	0.30	*TGFB1*, *BAX*
			0.21–19.72*	0.45	
22q11.1-q13.33	Loss	14.50–49.57	14.50–49.57*	0.45	*NF2*, *CHEK2*

Selected copy number gains/losses of ≥25% frequency (occurring in at least 5 out of 20 cases from unique patients) are listed with details pertaining to cytogenetic loci, genomic position, frequency, and candidate oncogenes or tumor suppressor genes. Involved maximal ranges and smaller subranges are included (coordinates in megabases).

* = ranges with cases showing slight variations within the interval.

**Figure 3:**
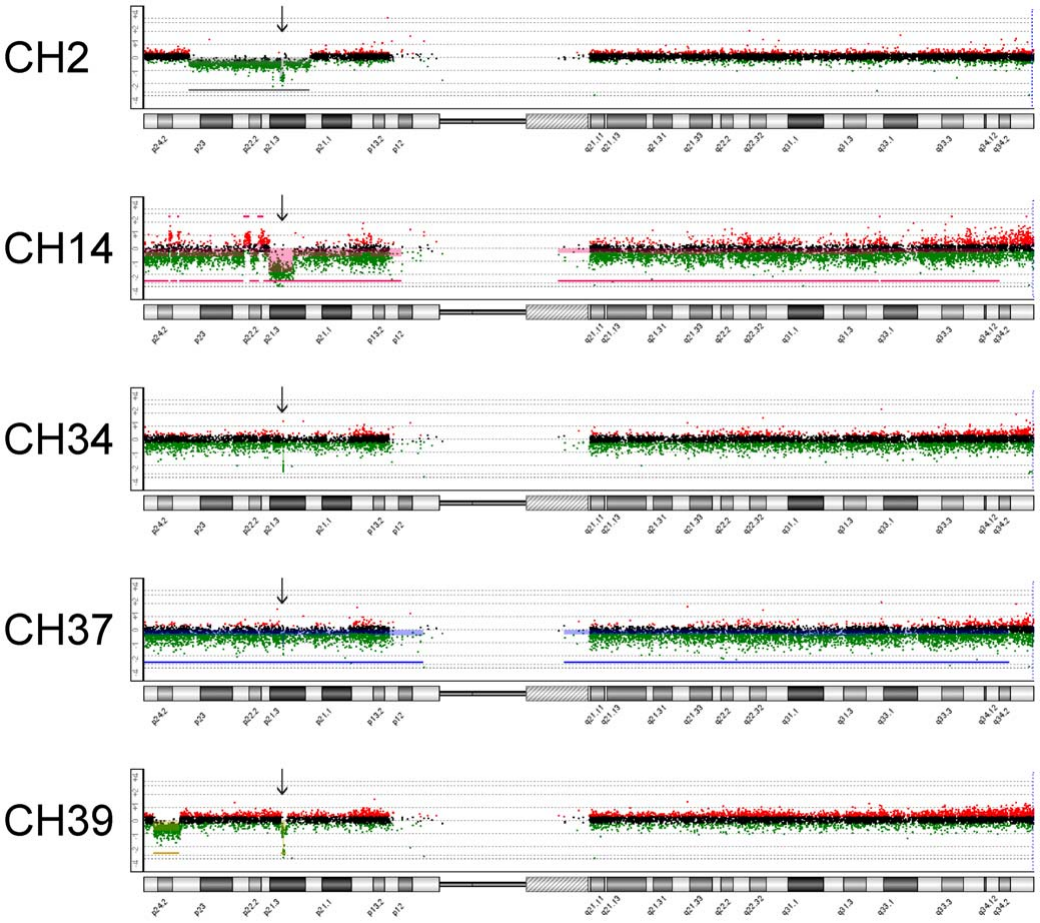
Chromosome 9 array CGH results. Array CGH results for chromosome 9 are shown for select chordoma cases showing homozygous *CDKN2A* deletion. Plots were generated with the Agilent Genomic Workbench Standard Edition 5.0 software. The sizes of the homozygous deletions for the five respective cases are as follows: 512 kb, 4.7 Mb, 76 kb, 76 kb, and 158 kb. Note that cases CH34 and CH37 are recurrent tumors from the same patient. Cases CH7 (158 kb) and CH36 (1.9 Mb) also harbor homozygous *CDKN2A* deletions and are not depicted above. kb = kilobases, Mb = megabases.

Loss of 9p, either through entire chromosome 9 loss or partial 9p loss alone, was observed in 15/20 cases (75%). Including one case with a submicroscopic deletion of approximately 158 kilobases involving *CDKN2A* only (CH7), 16 of 20 cases (80%) demonstrated loss of the *CDKN2A/CDKN2B* gene, six (30%) of which had a homozygous deletion of the gene (CH2, CH7, CH14, CH34, CH36, and CH39). Various patterns of homozygous *CDKN2A*/*CDKN2B* deletions were noted (Figure 3). Other homozygous submicroscopic deletions were detected by array CGH in regions of known benign copy number variation (CNV). Homozygous deletions of other pertinent tumor suppressor genes were not observed.

### CDKN2A Immunohistochemistry and PTEN Immunofluorescence

**Figure 4:**
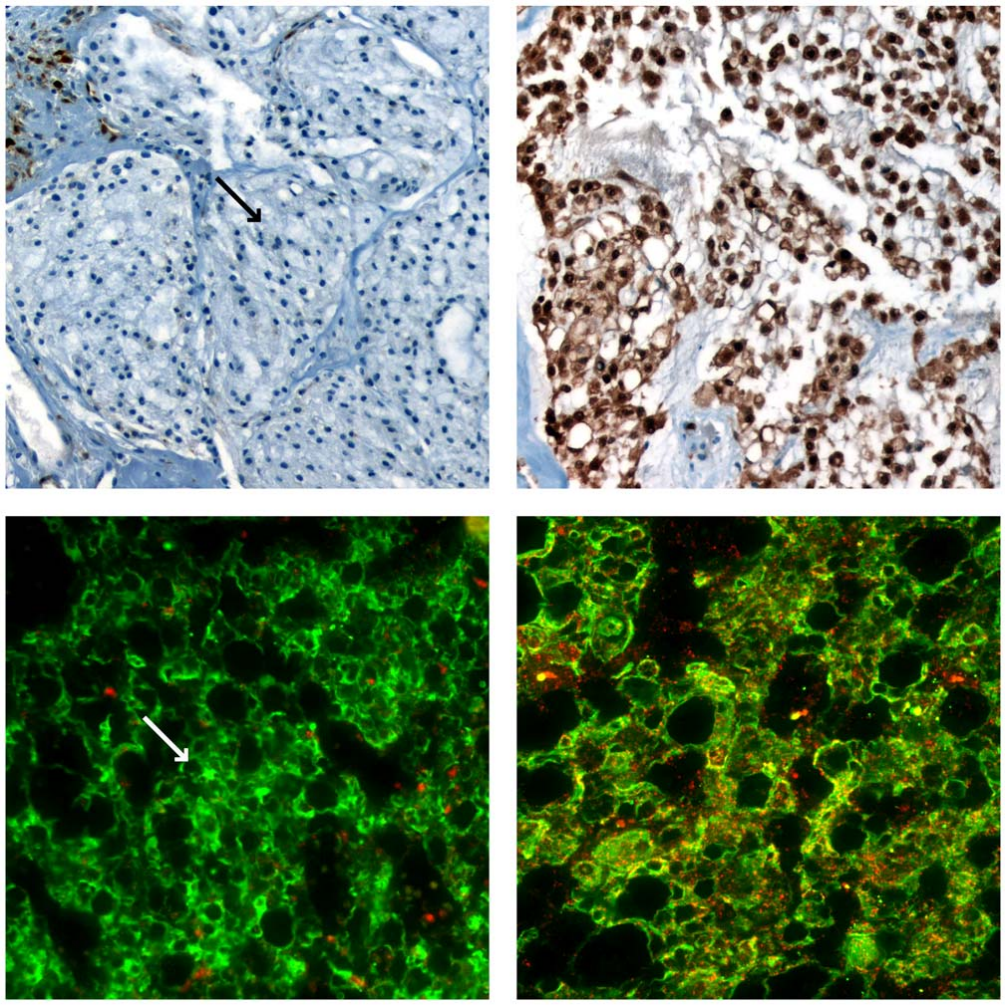
Top Panels: CDKN2A immunohistochemistry. Representative chordoma cases showing lack of expression (left, CH39) and strong expression (right, CH35). Bottom Panels: PTEN Immunofluorescence. Representative chordoma cases were immunostained with anti-cytokeratin to highlight tumor cells (green) and anti-PTEN (red). Tumor cells show lack of PTEN expression in the left panel and expression of PTEN in the right panel. Note in both left panels that stromal tissue or normal cells show expression of CDKN2A and PTEN, but tumor areas indicated by arrows show lack of expression.

The frequent copy number losses of 9p and 10q in our cohort of tumors directed our investigation into the loss of gene expression of key tumor suppressor genes found in these two regions. We performed protein immunostaining against CDKN2A and PTEN, two important tumor suppressor proteins found in 9p and 10q, respectively. Immunohistochemistry for CDKN2A showed loss of expression in the majority of cases (15/18 or 83% of cases tested, excluding CH37). Most of these cases demonstrated one or two copy number loss of *CDKN2A* on array CGH. All of the tested tumors with detected homozygous loss of *CDKN2A* on array CGH showed loss of CDKN2A protein expression (CH2, CH14, CH34, CH36, CH37, CH39) (Figure 4, top panels, and Table 3). Interestingly, two cases with maintenance of two *CDKN2A* copies also showed loss of CDKN2A expression (CH3 and CH33, italicized and bolded in Table 3).

**Table 3:** *CDKN2A* analysis results.

	CDKN2A	*CDKN2A*	*CDKN2A*	*CDKN2A*
Case	IHC	MSP1	MSP2	Copy Number Status
CH1	+	NP	NP	1
CH2	−	U	F	0
***CH3***	***−***	***NP***	***NP***	***2***
CH5	−	NP	NP	1
CH6	+	NP	NP	1
CH7	NP	U	U	0
CH8	NP	NP	NP	2
CH9	−	NP	NP	1
CH14	−	U	U	0
CH30	−	U	U	1
***CH33***	***−***	***M***	***M***	***2***
CH34	−	U	U	0
CH35	+	U	U	1
CH36	−	U	F	0
CH37	−	U	U	0
CH39	−	U	U	0
P527	−	U	U	1
P554	−	U	U	1
P937	−	U	U	1
P984	−	M	U	1
P1033	−	U	U	1

Immunohistochemistry (IHC) was performed using standard protocol on formalin-fixed paraffin-embedded tumor specimen to assess for expression of CDKN2A (9p21.3). Methylation specific PCR was performed on bisulfite treated DNA using two primers sets (MSP1 and MSP2) targeted to the promoter region of *CDKN2A*. *CDKN2A* copy number status for each case is summarized from the array CGH results. Italicized and bolded are two cases (CH3 and CH33) showing loss of CDKN2A while maintaining normal *CDKN2A* copy number status. NP = not performed. F = Technical failure. U = Unmethylated. M = Methylated. *CDKN2A* copy number status based on array CGH: 0 = Homozygous deletion, 1 = Hemizygous deletion, 2 = Copy number neutral.

Automated quantitative analysis (AQUA) of *in situ* PTEN expression using double immunofluorescence was utilized [16]. Anti-cytokeratin antibody, known to be a consistent marker for conventional and chondroid chordoma [20], was used to label the tumor cells green (AlexaFluor 555, Figure 4, bottom panels) and to create a tumor specific mask within which red anti-PTEN signals were automatically quantitated (CY5, Figure 4, bottom panels). PTEN immunofluoresence demonstrated a typical punctate nuclear and cytoplasmic staining pattern. Average pixel intensities with associated standard deviations were used to cluster the tested tumors into two distinct positive and negative staining groups by applying k-means clustering (Table 4). A non-parametric Mann-Whitney *U* test showed that these two groups are significantly different (median for positive staining group: 641, median for negative staining group: 108, *U* = 84, *n*_1_ = 6, *n*_2_ = 14, *P*<0.0001, two-tailed). The results indicate that most tumors with one copy loss of *PTEN* had loss of PTEN expression (11/16, 69%) while three out of four cases with maintenance of two *PTEN* copies showed loss of PTEN expression. Interestingly, the second recurrent CH37 tumor but not the first recurrent CH34 chordoma showed loss of PTEN expression. No significant correlation was noted between patient death and copy number status of *CDKN2A/B* or *PTEN*, or expression of CDKN2A/B or PTEN.

**Table 4:** *PTEN* analysis results.

	PTEN	*PTEN*	*PTEN*	*PTEN*
Case	IF	MSP1	MSP2	Copy Number Status
***CH1***	***−***	***NP***	***NP***	***2***
CH2	−	F	F	1
CH3	−	NP	NP	1
CH5	−	NP	NP	1
CH6	−	NP	NP	1
CH7	+	U	U	1
CH8	NP	NP	NP	1
CH9	+	NP	NP	1
CH14	−	U	U	1
CH30	+	U	U	2
***CH33***	***−***	***U***	***U***	***2***
CH34	+	U	M	1
CH35	−	U	U	1
***CH36***	***−***	***U***	***M***	***2***
CH37	−	U	M	1
CH39	−	U	U	1
P527	−	U	M	1
P554	−	U	U	1
P937	+	U	U	1
P984	−	U	M	1
P1033	+	U	U	1

Immunofluorescence (IF) was performed on formalin-fixed paraffin-embedded tumor specimen to assess for expression of PTEN (10q23.31) using the previously described AQUA technique (see Materials & Methods). Methylation specific PCR was performed on bisulfite treated DNA using two primers sets (MSP1 and MSP2) targeted to the promoter region of *PTEN*. *PTEN* copy number status for each case is summarized from the array CGH results. Italicized and bolded are three cases (CH1, CH33, and CH36) showing loss of PTEN despite maintainence of normal *PTEN* copy number status. NP = Not performed. F = Technical failure. U = Unmethylated. M = Methylated. *PTEN* copy number status based on array CGH: 0 = Homozygous deletion, 1 = Hemizygous deletion, 2 = Copy number neutral.

### CDKN2A and PTEN Methylation Specific PCR

**Figure 5:**
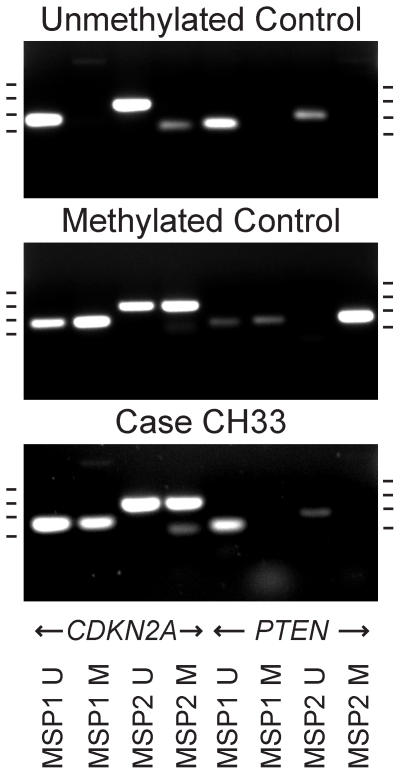
*CDKN2A* and *PTEN* methylation specific PCR. Bisulfite-treated chordoma DNA samples were tested with methylation specific PCR to evaluate for hypermethylation of the *CDKN2A* and *PTEN* promoter regions. Two sets of unmethylated (U) and methylated (M) PCR primers were used for each target gene (bottom labels, MSP1 and MSP2). Unmethylated and methylated controls are shown along with results for case CH33. Results for other tested cases are summarized in Tables 3 and 4 under the *CDKN2A* and *PTEN* MSP1 and MSP2 columns. Tick marks on the left and right of each panel indicate 100, 200, 300, and 400 base pair sizes (bottom to top).

*CDKN2A* and *PTEN* promoter hypermethylation was evaluated by using methylation specific PCR (MSP). Two sets of previously reported unmethylated and methylated specific primers were used in a touchdown PCR protocol to amplify CpG islands in the promoter regions after bisulfite treatment of the genomic DNA [18], [19]. Only one tested chordoma case (CH33) showed definitive evidence of *CDKN2A* promoter methylation with positive PCR amplification products using both sets of methylation specific primers (Table 3 and Figure 5). Equivocal *PTEN* promoter methylation specific PCR results (positive amplification with only one out of two primer sets) were obtained for five tested chordoma cases (CH34, CH36, CH37, P527, and P984, Table 4). No tumors showed definitive *PTEN* promoter methylation.

### Genotyping

A previously described multiplex single base extension genotyping assay (based on Life Technologies/Applied Biosystems SNaPshot technology) was applied to detect common point mutations in various cancer genes [17]. In total, fifty-six bases in various loci of the following genes were interrogated: *APC*, *CTNNB1*, *BRAF*, *EGFR*, *FLT3*, *JAK2*, *KIT*, *KRAS*, *NOTCH1*, *NRAS*, *PIK3CA*, *PTEN*, and *TP53*. No mutations in these common cancer genes were found in any of the 21 tumor samples (data not shown). *PTEN* exon 8 was further evaluated by reverse direction Sanger sequencing which also revealed no mutations (data not shown).

### T (Brachyury) Quantitative Real-Time PCR

**Figure 6:**
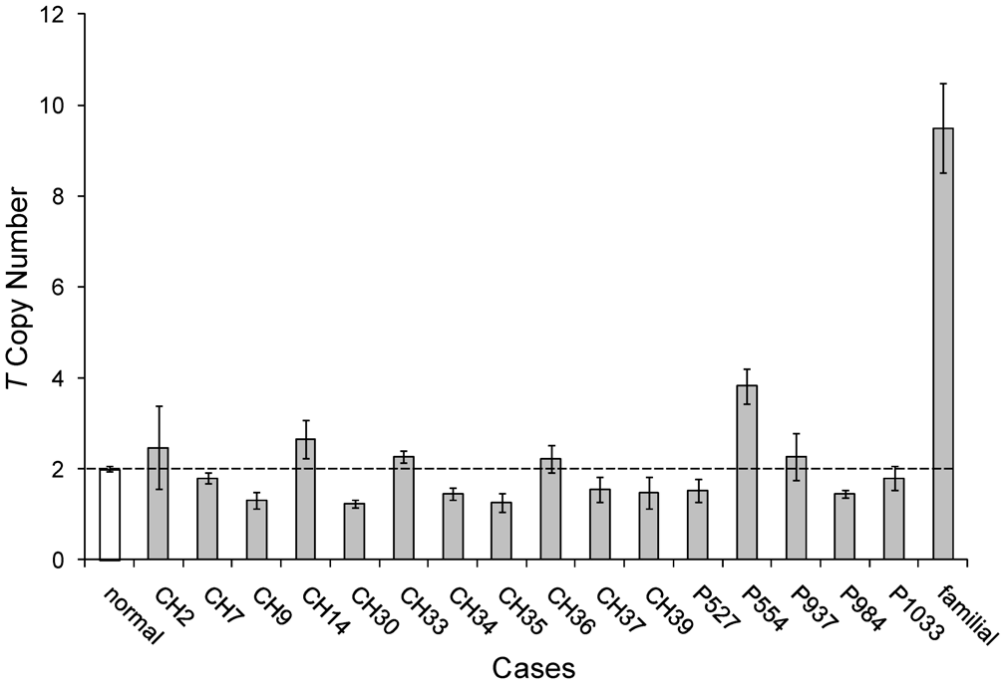
*T* quantitative real-time PCR. Two primer/probe sets were used to quantitate *T* (6q27) and *MCM7* (7q21.3-q22.1) (n = 3). Relative *T*∶*MCM7* ratios were normalized against an average ratio established from ten non-chordoma DNA samples (normal). The normalized ratios were corrected for *MCM7* copy number from array CGH data and approximate tumor percentage based on histological review. Corrected normalized ratios were multiplied by 2 to obtain the absolute *T* copy number. The dashed line represents a normal copy number of 2.

The identification of germline *T* gene duplication in familial chordomas [10] prompted us to perform quantitative real-time PCR in our set of sporadic chordomas using one set of primer/probe targeted to *T* and a reference control primer/probe set targeted to *MCM7*. Relative *T*∶*MCM7* ratios were determined and normalized against an average ratio established from a normal control run of 10 non-chordoma genomic DNA samples (Figure 6). All but two tested sporadic chordoma samples (CH14 and P554) showed a normal *T* copy number of 2. A *T* copy number of 2.7±0.4 was determined for case CH14 and 3.8±0.4 for case P554. A familial case of chordoma (family 4, patient 1) previously reported to have *T* duplication [10], showed a *T* copy number of approximately 9.5±1.0.

## Discussion

Using the Agilent 244K genome wide CGH microarray, we found numerous albeit recurrent and remarkably stereotypic chromosomal abnormalities in sporadic chordomas. In aggregate, the changes characterize a malignancy with significant genomic instability. Copy number losses were more prevalent than copy number gains, specifically 1p which was partially or completely lost in all of our chordoma cases. Prior studies examining 1p have described similar results, implicating in particular loss of the 1p36 locus as a candidate chromosomal region in both the development of chordoma and as a poor prognostic factor [15], [21], [22], [23]. An important tumor suppressor gene located in this locus is *RUNX3* which is often deleted or hypermethylated in various cancers showing epithelial, hematopoietic, and neural phenotypes [24]. Eighteen of twenty (90%) chordoma cases showed hemizygous loss of 1p36, suggesting the relevance of examining *RUNX3* as well as other potential 1p36 tumor suppressor genes in chordomas in future studies (e.g., *CDH5*, *TP73*, or *CDKN2C*).

Other common observed losses include chromosomes 3, 4, 9p, 9q, 10, 13, 14, 18, and 22. Common gains include chromosomes 7 and 19. These results mirror those found previously by Hallor *et al.* although the frequencies of these changes in our study range from 10% to 30% more than in their report [15]. Similar to the work of Hallor *et al.*, the only consistent homozygous deletion that was detected in our 21 chordoma cases involves 9p and specifically the *CDKN2A/CDKN2B* genes. The loss of 9p is an established finding in chordomas based on experiments involving FISH [8], [25] and BAC array CGH [15]. The CDKN2A protein or p16, which is encoded by the *CDKN2A* gene on the short arm of chromosome 9 (9p21), is a tumor suppressor gene that inhibits the function of cdk4- and cdk6-cyclin D complexes. Cdk-cyclin complexes regulate the retinoblastoma protein, thus controlling the G1-S phase checkpoint of the cell cycle. *CDKN2A* inactivation thereby can result in cellular proliferation [26].

Review of our array CGH data showed various mechanisms of homozygous loss of *CDKN2A* only or *CDKN2A*/*CDKN2B* in 30% of our cases (excluding CH37), including submicroscopic deletions in the setting of 9p one copy loss (CH2, CH7, CH34, CH36, and CH37), submicroscopic deletions alone (CH39), or deletions as part of complex changes in 9p (CH14) (Figure 3). The homozygous deletions ranged from approximately 76 kilobases to 4.7 megabases. Interestingly, both the first and second recurrent tumors from the same patient (CH34 and CH37, respectively) shared the same affected probes in their homozygous copy loss, spanning about 76 kb and involving only the *CDKN2A* gene. Of note, the complex “saw-tooth,” alternating gain-loss pattern observed with sample CH14 may reflect a similar pattern found in a recently reported chordoma case showing a catastrophic rearrangement phenomenon called “chromothripsis,” which also involved chromosome 9p [27].

The frequencies at which we detected hemizygous and homozygous deletions of the *CDKN2A* locus were overall higher than those described by Hallor *et al.*: 10/20 or 50% hemizygous and 6/20 or 30% homozygous (16/20 or 80% total) versus 15/26 or 58% hemizygous and 3/26 or 12% homozygous (18/26 or 69% total). The difference in pickup rate may be attributed to our use of a higher resolution Agilent 244K oligo array which affords higher sensitivity in detecting smaller changes relative to the traditional techniques applied in the Hallor *et al.* study, including karyotyping, FISH, and BAC array CGH. There was no statistically significant association between death and the presence of hemizygous or homozygous 9p (containing *CDKN2A*) loss (Fisher's exact test, *P* = 0.59, 2-tail) or the presence of homozygous 9p (containing *CDKN2A*) loss alone (Fisher's exact test, *P* = 1, 2-tail).

Array CGH findings related to *CDKN2A* were correlated with protein expression. Indeed immunohistochemistry for CDKN2A confirmed loss of expression in 15/18 or 83% (excluding CH37) of tested cases, including 8/11 tumors with hemizygous deletion, 5/5 tumors with homozygous deletion, and 2/2 tumors with copy number maintenance. Other studies have also illustrated similar results with regard to loss of CDKN2A expression [28], [29]. No statistically significant association between death and CDKN2A immunohistochemistry was found (Fisher's exact test, *P* = 0.56, 2-tail). In tumors with hemizygous deletions, loss of expression of the remaining allele through promoter methylation of *CDKN2A* may eliminate expression from the second non-deleted allele. *CDKN2A* promoter methylation analysis using two sets of methylation specific primers showed definitive promoter methylation in only one case (CH33) which explains the loss of CDKN2A expression despite maintaining 2 copies of *CDKN2A*. These results indicate promoter methylation may be a mechanism of *CDKN2A* gene silencing in only a small subset of chordomas.

The *PTEN* tumor suppressor gene is located on 10q23.3, a region which showed hemizygous deletions in 80% of our chordoma samples. No statistically significant association between death and 10q (including *PTEN*) copy number status was found (Fisher's exact test, *P* = 0.09, 2-tail). Quantitative immunofluorescence was applied to evaluate the expression of PTEN, showing widespread loss of expression observed in 13/19 or 68% of the tested tumors (excluding CH37). Three of these cases (CH1, CH33, and CH36) had normal copy number status of the gene. Interestingly, loss of PTEN expression was acquired in the second recurrent CH37 tumor but not in the first recurrent CH34 chordoma of the same patient. Loss of PTEN expression was not associated with patient death (Fisher's exact test, *P* = 1, 2-tail). The frequent loss of PTEN expression is consistent with our recent study describing hyperactivation of Akt/mTORC1 signaling in sporadic sacral chordomas as a result of PTEN deficiency [30].

Methylation analysis of 4/5 tested cases with intact PTEN expression did not show methylation of the *PTEN* promoter region. In 5/15 tested cases (including CH34 which had intact PTEN expression), equivocal *PTEN* methylation specific PCR results were obtained in which only the MSP2 primer set amplified the methylated target at nucleotide −298 upstream in the promoter sequence while the MSP1 primer set targeting nucleotide −984 did not. Both primer sets were reported to be applicable in assessing *PTEN* promoter methylation status in a manner specific to the putative gene rather than the pseudogene [19]. It is possible that locus heterogeneity of CpG island methylation may explain these results, a phenomenon which has been reported for *MGMT* promoter methylation in glioblastomas [31]. Without functional studies to characterize the methylation effect of individual CpG islands on protein expression, our current findings cannot definitively confirm or refute the role of promoter methylation in the silencing of PTEN expression in sporadic chordomas. However, if these five equivocal cases were truly methylated, *PTEN* promoter methylation would account for 4/9 tested cases with loss of PTEN expression and therefore not be the basis for gene silencing in the majority of chordoma cases with PTEN deficiency.

We also explored whether genetic mutations may account for the loss of PTEN expression/function. Our single base extension genotyping SNaPshot platform (examining the common PTEN R130*, R173C, R233*, K267fs*9 mutations) and Sanger sequencing of *PTEN* exon 8 did not reveal any mutations in the tested chordoma samples. Our SNaPshot assay also includes primers to evaluate hotspot point mutations in other genes commonly found in cancer including *APC*, *CTNNB1*, *BRAF*, *EGFR*, *FLT3*, *JAK2*, *KIT*, *KRAS*, *NOTCH1*, *NRAS*, *PIK3CA*, and *TP53*. No mutations in these genes were detected. Our negative *KRAS*/*BRAF* results are similar to the negative findings of another study which tested chordomas for *KRAS* and *BRAF* mutations and their relationship with the FGFR-RAS/RAF/MEK/ERK-ETS2/brachyury pathway [32].

The recent implication of *T* (brachyury) gene duplication in familial chordoma [10] and *T* copy number gain in sporadic chordoma [11] prompted us to search for a possible association in our group of sporadic chordomas. The findings from the work of Yang *et al.* suggested that the duplicated *T* region in familial chordoma can range from 52 kb to 489 kb. The Agilent 244K microarray used in this study contains only two probes in the *T* gene which are spaced 8216 bases apart (hg18) and are therefore inadequate for evaluating *T* duplication. To more accurately assess the copy number of *T* in our sporadic chordoma samples, we applied quantitative real-time PCR. The data suggest that unlike familial chordomas, the majority of our sporadic chordoma cases do not possess *T* duplication or amplification. Only two out of 16 analyzed cases (CH14 and P554) showed an abnormal *T* copy number (approximately 3 and 4 copies per cell, respectively). A familial chordoma sample (family 4, patient 1), which was tested in the Yang *et al.* study and served as our positive control, showed amplification at nearly 10 copies per cell. Our results are in line with the recent Shalaby *et al.* study which utilized FISH and found no *T* amplification in the majority of their 39 tested sporadic chordoma cases and a minor *T* allelic gain (at approximately a 3∶1 ratio) in 3 samples [32]. In addition, Presneau *et al.* also showed *T* amplification in 14 of 181 and minor allelic gain in 8 of 81 sporadic chordoma cases [11]. Our two cases which showed *T* copy number gains (CH14 and P554) are consistent with the minor *T* allelic gain observation in both the Shalaby *et al.* and Presneau *et al.* studies.

In summary, we have shown that sporadic chordoma is a malignant disease characterized by significant genomic instability mostly due to large copy number losses. In addition to validating previously reported cytogenetic findings from other studies, we identified smaller, recurrent homozygous deletions in the *CDKN2A/CDKN2B* locus. The frequent loss of chromosomal regions containing *CDKN2A* and *PTEN* tumor suppressor genes was associated with loss of protein expression which mechanistically does not appear to be due to promoter methylation in the majority of cases and did not correlate with patient death. Sporadic chordomas do not harbor point mutations in some of the common cancer genes and are not associated with *T* duplication or amplification in most cases. The findings indicate that the majority of sporadic chordomas may rely on mechanisms other than copy number gain if they indeed exploit T/brachury for proliferation. Future studies should utilize high-throughput genotyping methods such as next generation sequencing to account for loss of heterozygosity in *CDKN2A* and *PTEN*, examine the role of *T* in proliferation, and search for mutations outside of known hotspots in cancer genes.
